# Withdrawal of inhaled corticosteroids versus continuation of triple therapy in patients with COPD in real life: observational comparative effectiveness study

**DOI:** 10.1186/s12931-021-01615-0

**Published:** 2021-01-21

**Authors:** Helgo Magnussen, Sarah Lucas, Therese Lapperre, Jennifer K. Quint, Ronald J. Dandurand, Nicolas Roche, Alberto Papi, David Price, Marc Miravitlles, the Respiratory Effectiveness Group (REG)

**Affiliations:** 1 Pulmonary Research Institute at Lung Clinic Grosshansdorf, Airway Research Center North, Member of the German Center of Lung Research, Grosshansdorf, Germany; 2 Respiratory Effectiveness Group, Ely, UK; 3 Department of Respiratory Medicine, Antwerp University Hospital, Edegem, Denmark; 4 Laboratory of Experimental Medicine and Paediatrics, University of Antwerp, Wilrijk, UK; 5 CIUSSS de L’Ouest-de-L’Île-de-Montréal, Montreal Chest Institute, Meakins-Christie Laboratories, Oscillometry Unit and Centre for Innovative Medicine, McGill University Health Centre and Research Institute, Montreal, QC Canada; 6 Department of Respiratory Medicine, APHP-Centre University of Paris, UMR1016, Cochin Institute, Paris, France; 7 Section of Cardiorespiratory and Internal Medicine, Department of Medical Sciences, University of Ferrara, Ferrara, Italy; 8 Observational and Pragmatic Research Institute, Singapore, Singapore; 9 Centre of Academic Primary Care, Division of Applied Health Sciences, University of Aberdeen, Aberdeen, UK; 10 Pneumology Department, Hospital Universitari Vall D’Hebron, Vall D’Hebron Institut de Recerca (VHIR), CIBER de Enfermedades Respiratorias (CIBERES), Vall d’Hebron Barcelona Hospital Campus, Passeig Vall d’Hebron 119-129, 08035 Barcelona, Spain

**Keywords:** COPD, Inhaled corticosteroids, Withdrawal, Real life, Effectiveness

## Abstract

**Background:**

Inhaled corticosteroids (ICS) are indicated for prevention of exacerbations in patients with COPD, but they are frequently overprescribed. ICS withdrawal has been recommended by international guidelines in order to prevent side effects in patients in whom ICS are not indicated.

**Method:**

Observational comparative effectiveness study aimed to evaluate the effect of ICS withdrawal versus continuation of triple therapy (TT) in COPD patients in primary care. Data were obtained from the Optimum Patient Care Research Database (OPCRD) in the UK.

**Results:**

A total of 1046 patients who withdrew ICS were matched 1:4 by time on TT to 4184 patients who continued with TT. Up to 76.1% of the total population had 0 or 1 exacerbation the previous year. After controlling for confounders, patients who discontinued ICS did not have an increased risk of moderate or severe exacerbations (adjusted HR: 1.04, 95% confidence interval (CI) 0.94–1.15; p = 0.441). However, rates of exacerbations managed in primary care (incidence rate ratio (IRR) 1.33, 95% CI 1.10–1.60; p = 0.003) or in hospital (IRR 1.72, 95% CI 1.03–2.86; p = 0.036) were higher in the cessation group. Unsuccessful ICS withdrawal was significantly and independently associated with more frequent courses of oral corticosteroids the previous year and with a blood eosinophil count ≥ 300 cells/μL.

**Conclusions:**

In this primary care population of patients with COPD, composed mostly of infrequent exacerbators, discontinuation of ICS from TT was not associated with an increased risk of exacerbation; however, the subgroup of patients with more frequent courses of oral corticosteroids and high blood eosinophil counts should not be withdrawn from ICS.

*Trial registration* European Network of Centres for Pharmacoepidemiology and Pharmacovigilance (EUPAS30851).

## Background

Pharmacological therapy for COPD is directed to reduce symptoms, reduce the frequency and severity of exacerbations, and improve exercise tolerance and health status [1]. The mainstay of pharmacological therapy is long-acting bronchodilators, either inhaled long-acting muscarinic antagonists (LAMAs), inhaled long-acting β2-agonists (LABAs), or the combination of both. Inhaled corticosteroids (ICS) can be added to LABA or to the combination of LABA and LAMA leading to triple therapy (TT) in patients with persisting exacerbations despite optimal bronchodilator treatment, particularly if they have high blood eosinophil counts and or history of asthma [1].

A comparison of dual bronchodilation versus TT has been performed predominately in randomised controlled trials (RCT), which have demonstrated the superiority of TT in particular in frequent exacerbators, patients at risk of hospital admission and those with higher concentrations of blood eosinophils [2, 3]. However, there is increasing evidence that patients recruited for RCTs may not completely reflect the characteristics of patients attending in primary care, and therefore, non-interventional, observational studies are important to confirm the findings of RCTs [4]. Regarding efficacy, a large observational study in the UK showed that TT was more effective than dual bronchodilation in preventing exacerbations in patients with increasing blood eosinophil counts and number of previous exacerbations, but not in patients with infrequent exacerbations and low blood eosinophils [5].

Despite the existing evidence and the current recommendations, there is frequent use of TT out of indication, both in primary and secondary care [6–9]. This overuse of ICS and the risks associated with their long-term use, have generated some consensus statements on ICS recommendations for ICS withdrawal [10–15]. These recommendations are based on RCTs, but again, since a significant number of patients in primary care may not be represented in RCTs, it is important to also investigate the possible impact of ICS withdrawal in usual clinical practice.

The current study has used data from a large administrative healthcare database in the UK to investigate the consequences of ICS withdrawal from TT with continuation on dual bronchodilation compared to continuation on TT in patients with COPD followed in primary care.

## Methods

### Study design and population

This was an observational comparative effectiveness study aimed to evaluate the effect of inhaled corticosteroid (ICS) cessation versus continuation of triple therapy in COPD patients.

Exacerbations, symptoms and lung function were compared for a period of 1 year after the index prescription date (IPD), the outcome year, between patients who withdrew and those who continued with ICS in the form of triple therapy. Characteristics of the patients and frequency of exacerbations were collected during the year prior to the IPD, or baseline year.

Patients were required to have ≥ 2 fixed dose ICS/LABA and separate LAMA prescriptions, or ≥ 2 fixed dose ICS/LABA/LAMA prescriptions, in the baseline year. The IPD for the cessation group was the first prescription for a single LABA alongside a single LAMA, or a fixed dose LABA/LAMA, without ICS.

**Fig. 1:**
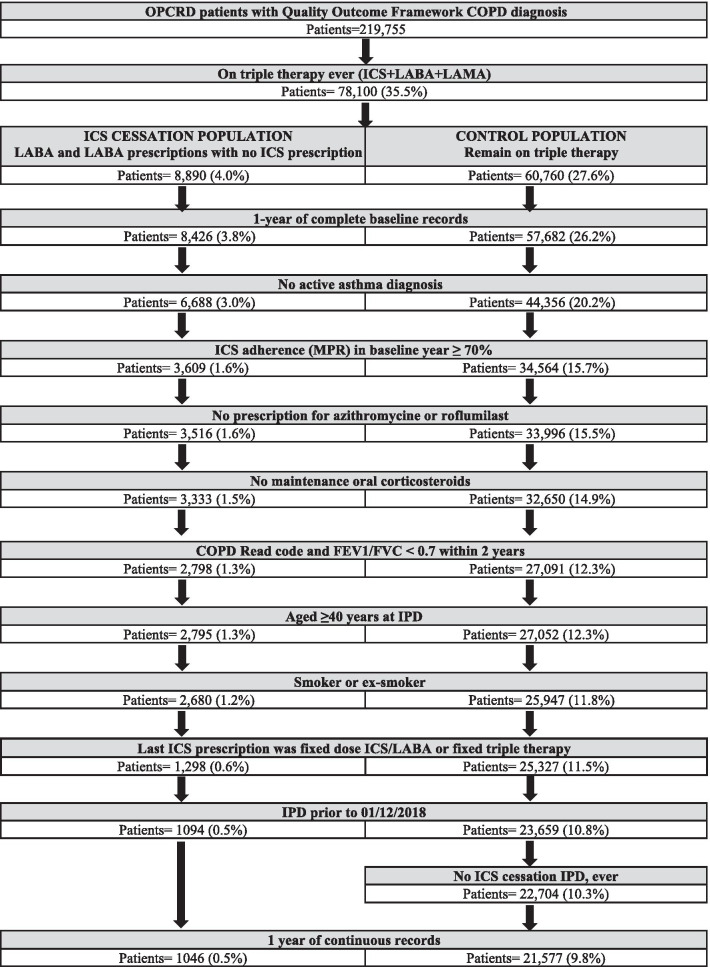
Patients’ flow chart

The control group patients were required to have ≥ 1 fixed or free combination of ICS/LABA/LAMA in the outcome year (Fig. 1). Their IPD was the date when the patient received a repeated prescription for their baseline triple therapy.

Patients were required to have an IPD prior to 1/12/2018 to allow for a 1-year outcome period; in patients with more than one IPD the first IPD was used for analysis.

Data were obtained from the Optimum Patient Care Research Database (OPCRD). The OPCRD contains anonymised, longitudinal medical records for nearly 9 million UK primary care patients, from more than 700 GP practices across the UK. The OPCRD is approved by the Trent Multi-Centre Research Ethics Committee for clinical research use. This study was approved by the Anonymised Data Ethics & Protocol Transparency committee (ADEPT1419) and registered with the European Network of Centres for Pharmacoepidemiology and Pharmacovigilance (EUPAS30851).

Inclusion criteria were: (A) spirometry-confirmed diagnosis of COPD (Read code and FEV_1_/FVC < 0.7 within 2 years, ever recorded); (B) aged ≥ 40 years at IPD; (C) current or ex-smoker; (D) have ≥ 1 year of continuous patient records in prior to IPD; and (E) ICS medication possession ratio (MPR, (Number of days supplied in period/Days in period) × 100) ≥ 70% in the baseline year.

Exclusion criteria were: (A) asthma Read code during the baseline year; (B) prescribed azithromycin or roflumilast or receiving maintenance treatment with systemic steroids. Patients were excluded from the control group if they had ever had an ICS cessation prior to IPD.

### Study outcomes

Outcomes were assessed in the 1-year period following IPD, the outcome year. The primary outcome was time to first COPD exacerbation. An exacerbation was defined as: an unscheduled hospital admission or A&E attendance for COPD/respiratory condition or generic hospitalisation code on the same day as a lower respiratory coded consultation, course of oral steroids and/or antibiotics prescribed with lower respiratory consultation. More than one oral steroid course, A&E attendance, hospitalisation or prescription for antibiotics occurring within 2 weeks of each other were considered the result of the same exacerbation and were only be counted once.

The secondary outcomes were: exacerbation rate, annualised change in FEV1, where baseline FEV1 was recorded anytime in the baseline year and outcome FEV1 was recorded between 9 and 15 months post IPD; COPD Assessment Test (CAT) score in outcome year; modified Medical Research Council (mMRC) dyspnoea score in the outcome year; time to first consultation with a pneumonia Read code.

### Statistical analysis

In order to select an IPD for the control patients and reduce the risk of survival bias, control patients were selected by matching 1:4 with ICS cessation patients based on time on triple therapy using optimal matching. Intention-to-treat analyses were performed using R software (www.r-project.org/).

Demographics and clinical characteristics were compared between the ICS cessation and control groups using chi-squared and Mann–Whitney U tests, as appropriate. For all outcomes univariate analyses of the following baseline variables were conducted: age, sex, Body Mass Index (BMI), comorbidities, GOLD stage, smoking status, blood eosinophils, CAT score, mMRC dyspnea score, pre-baseline asthma diagnosis, number of respiratory consultations, exacerbations managed in primary care, exacerbations requiring A&E attendance or hospitalisation in the baseline year and the numbers of ICS prescriptions, antibiotics prescriptions and oral corticosteroid prescriptions in the baseline year. The time to first exacerbation and time to first pneumonia were analysed using multivariate Cox proportional hazards regression. Exacerbation rate was analysed using multivariate negative binomial regression, change in FEV1 was analysed using multivariate linear regression and changes in CAT scores and mMRC dyspnea were analysed using multivariate logistic regression. Confounding was adjusted for with the use of multivariate analyses. Variables for inclusion in multivariate regression models were selected using fast backwards elimination.

In primary care databases mortality is not well recorded so as a proxy to explore any differences between the ICS cessation and control groups we initially assessed the time until patients left the database; patients may have been recorded as leaving as a result of death or moving to a new practice. No statistically significant difference in time to leaving the database was found between the two groups (Additional file 1: Figure S1), so those who left and lacked a year of continuous records were removed from subsequent analyses.

## Results

### Patients’ characteristics

The final dataset consisted of 1046 ICS cessation patients and 21,577 control patients, of whom 4184 were matched for time on triple therapy before IPD and constitute the population of our study (Fig. 1 and Additional file 1: Table S1). There were no significant differences between groups in age, sex distribution, time since COPD diagnosis and smoking status. Patients who had ICS withdrawn had a milder disease with a mean FEV1 (%) of 58.2% compared to 53.9% (p = 0.003), lower concentrations of blood eosinophils (p = 0.006) and had more respiratory consultations and pneumonia coded consultations in Primary Care in the baseline year (both p = 0.001). They also less commonly had a diagnosis of asthma before baseline: 14.1% versus 26.9% (p < 0.001). Patient demographics, comorbidities and healthcare and medication utilisation in the baseline year are presented in Tables 1 and 2.

**Table 1:** Patient demographics and clinical characteristics

	Total No. 5230	Control No. 4184	ICS cessation No. 1046	p-value
Age, years				
Mean (SD)	70.8 (± 9.9)	70.7 (± 10.2)	71.0 (± 8.8)	0.61
Sex				
Female	2330 (44.6%)	1872 (44.7%)	458 (43.8%)	0.60
Male	2900 (55.4%)	2312 (55.3%)	588 (56.2%)	
BMI category				
Mean (SD)	27.3 (± 7.6)	27.1 (± 7.3)	28.1 (± 8.7)	< 0.001
Missing	204 (3.9%)	181 (4.3%)	23 (2.2%)	
Smoking status				
Current smoker	1813 (34.7%)	1467 (35.1%)	346 (33.1%)	0.24
Ex-smoker	3417 (65.3%)	2717 (64.9%)	700 (66.9%)	
FEV_1_% predicted				
Mean (SD)	54.8 (± 22.2)	53.9 (± 22.5)	58.2 (± 20.9)	0.003
Missing	471 (9.0%)	417 (10.0%)	54 (5.2%)	
Blood eosinophil count				
< 0.1	370 (8.6%)	271 (8.0%)	99 (11.1%)	0.006
≥ 0.1 to < 0.3	2947 (68.7%)	2339 (68.8%)	608 (68.4%)	
≥ 0.3	970 (22.6%)	788 (23.2%)	182 (20.5%)	
Missing	943 (18.0%)	786 (18.8%)	157 (15.0%)	
CAT score				
Mean (SD)	16.8 (± 9.3)	17.1 (± 9.6)	16.0 (± 8.5)	0.057
Missing	4056 (77.6%)	3370 (80.5%)	686 (65.6%)	
mMRC dyspnea scale				
Mean (SD)	1.9 (± 1.0)	1.9 (± 1.0)	1.8 (± 0.9)	< 0.001
Missing	340 (6.5%)	300 (7.2%)	40 (3.8%)	
Time since first COPD diagnosis, year				
Mean (SD)	8.5 (± 6.7)	8.5 (± 6.8)	8.5 (± 6.2)	0.37
Missing	152 (2.9%)	124 (3.0%)	28 (2.7%)	
Comorbidities				
Asthma (diagnosed pre baseline)^a^	1273 (24.3%)	1126 (26.9%)	147 (14.1%)	< 0.001
Asthma (diagnosed in year post IPD)	18 (0.34%)	12 (0.29%)	6 (0.57%)	0.23
Bronchiectasis	368 (7.0%)	304 (7.3%)	64 (6.1%)	0.22
Active rhinitis	1146 (21.9%)	918 (21.9%)	228 (21.8%)	0.93
Nasal polyps	128 (2.4%)	105 (2.5%)	23 (2.2%)	0.65
Active GERD	2517 (48.1%)	1971 (47.1%)	546 (52.2%)	0.003
Cardiovascular disease	2358 (45.1%)	1892 (45.2%)	466 (44.6%)	0.70
Ischaemic heart disease	1161 (22.2%)	935 (22.3%)	226 (21.6%)	0.62
Heart failure	537 (10.3%)	444 (10.6%)	93 (8.9%)	0.11
Myocardial infarction	591 (11.3%)	472 (11.3%)	119 (11.4%)	0.91
Cerebrovascular disease	424 (8.1%)	347 (8.3%)	77 (7.4%)	0.34
Hypertension	2483 (47.5%)	1961 (46.9%)	522 (49.9%)	0.083
Diabetes	1040 (19.9%)	837 (20.0%)	203 (19.4%)	0.70
Osteoporosis	682 (13.0%)	553 (13.2%)	129 (12.3%)	0.47
Anxiety and/or depression	2341 (44.8%)	1871 (44.7%)	470 (44.9%)	0.92
Chronic kidney disease	861 (16.5%)	695 (16.6%)	166 (15.9%)	0.58
Other chronic diseases	920 (17.6%)	768 (18.4%)	152 (14.5%)	0.004

^a^Resolved or Read code prior to, but not during, baseline year

**Table 2:** Healthcare and medication utilisation in the baseline year

	Total No. 5230	Control No. 4184	ICS cessation No. 1046	p-value
Respiratory consultations in primary care	3.8 (± 3.6)	3.7 (± 3.4)	4.1 (± 4.0)	0.001
Exacerbations managed in primary care	1.0 (± 1.4)	1.0 (± 1.4)	1.0 (± 1.3)	0.41
Exacerbations requiring A&E attendance or hospitalisation	0.08 (± 0.32)	0.08 (± 0.31)	0.09 (± 0.35)	0.21
Total exacerbations	1.1 (± 1.4)	1.1 (± 1.4)	1.1 (± 1.4)	0.68
Pneumonia-coded consultation, n (%)	153 (2.9%)	101 (2.4%)	52 (5.0%)	< 0.001
ICS prescriptions	11.1 (± 3.7)	10.9 (± 3.5)	12.3 (± 4.3)	< 0.001
ICS adherence (medication possession ratio)	98.4 (± 37.9)	96.1 (± 37.5)	107.8 (± 38.2)	< 0.001
LABA prescriptions	11.2 (± 4.2)	10.8 (± 3.8)	12.6 (± 5.1)	< 0.001
LAMA prescriptions	9.6 (± 4.7)	9.3 (± 4.6)	11.1 (± 4.8)	< 0.001
SABA prescriptions	11.4 (± 9.9)	11.4 (± 9.7)	11.6 (± 10.5)	0.66
Theophylline prescriptions, n (%)	440 (8.4%)	384 (9.2%)	56 (5.4%)	< 0.001
Carbocysteine prescriptions, n (%)	1142 (21.8%)	920 (22.0%)	222 (21.2%)	0.62
Antibiotic prescriptions, n (%)	2571 (49.2%)	2078 (49.7%)	493 (47.1%)	0.15
Oral corticosteroid prescriptions, n (%)	3122 (59.7%)	2552 (61.0%)	570 (54.4%)	< 0.001

Data are means (± standard deviation), unless otherwise specified

### Primary outcome: time to first COPD exacerbation

**Fig. 2:**
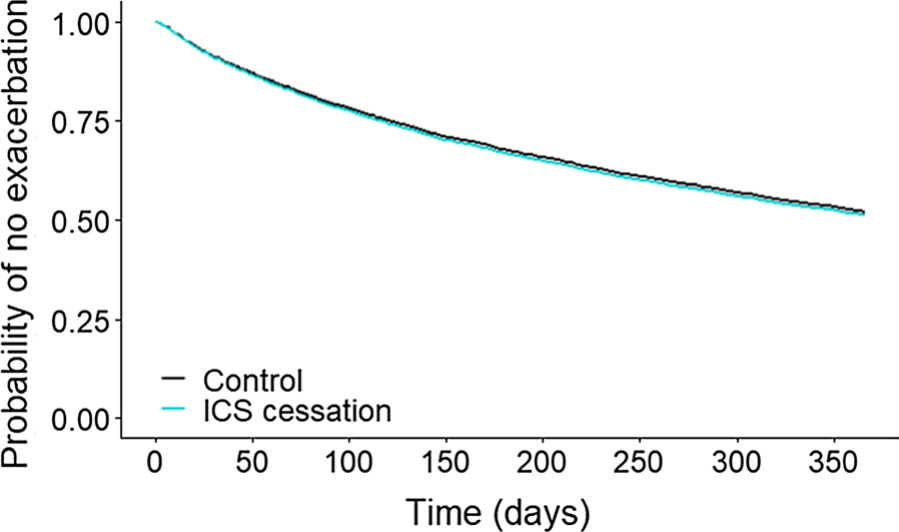
Plot of multivariate Cox proportional hazards model of time to first exacerbation in the 1-year outcome period. HR = 1.04 (95% CI 0.94–1.15; p = 0.441)

The cessation of ICS was not associated with an increased risk of having an exacerbation in the outcome year both in univariate (hazard ratio (HR) 1.02 (95% confidence interval (CI) 0.92–1.12) and in multivariate analysis controlling for confounders (HR 1.04 (95% CI 0.94–1.15), p = 0.441) (Table 3, Fig. 2). Only number of exacerbations managed in primary care and oral corticosteroids prescriptions in baseline year were significantly associated with a reduced time to the first exacerbation in multivariate Cox regression analysis (Table 3).

**Table 3:** Cox proportional hazards regression analysis evaluating the effect of ICS cessation and variables associated with the time to first exacerbation in the 1-year outcome period

Variable	Total	Event	Univariate		Multivariate		p-value
			HR	95% CI	HR	95% CI	
Group							
Control	4184	2008 (47.9%)	1	Ref			
ICS cessation	1046	501 (47.9%)	1.02	0.92 to 1.12	1.04	0.94 to 1.15	0.441
Sex							
Female	2330	1190 (51.1%)	1	Ref			
Male	2900	1319 (45.5%)	0.85	0.79 to 0.92			
GOLD severity category							
Mild	525	236 (44.9%)	1	Ref			
Moderate	2173	1019 (46.9%)	1.05	0.91 to 1.21			
Severe	1576	790 (50.1%)	1.15	0.99 to 1.33			
Very severe	485	258 (53.2%)	1.26	1.06 to 1.51			
Blood eosinophil count							
< 0.1	370	173 (46.7%)	1	Ref			
≥ 0.1 to < 0.3	2947	1452 (49.3%)	1.07	0.91 to 1.25			
≥ 0.3	970	490 (50.5%)	1.1	0.93 to 1.31			
CAT score							
0–9	275	108 (39.2%)	1	Ref			
10–19	457	206 (45.1%)	1.2	0.95 to 1.52			
20–29	318	155 (48.7%)	1.33	1.04 to 1.70			
30–40	124	74 (59.7%)	1.82	1.35 to 2.44			
mMRC dyspnea scale							
0	292	129 (44.2%)	1	Ref			
1	1538	686 (44.6%)	1.04	0.86 to 1.25			
2	1648	830 (50.3%)	1.2	1.00 to 1.44			
3	1168	579 (49.6%)	1.19	0.98 to 1.44			
4	244	124 (50.8%)	1.23	0.96 to 1.57			
Asthma diagnosis pre-baseline							
No	3957	1864 (47.1%)	1	Ref			
Yes	1273	645 (50.6%)	1.13	1.03 to 1.23			
Respiratory consultations in baseline year							
0	179	52 (29.0%)	1	Ref			
1–2	2116	777 (36.7%)	1.33	1.00 to 1.76			
3–4	1434	677 (47.2%)	1.84	1.39 to 2.44			
5+	1501	1003 (66.8%)	3.33	2.52 to 4.40			
Exacerbations managed in primary care in baseline year							
0	2573	851 (33.1%)	1	Ref			
1	1404	732 (52.1%)	1.81	1.64 to 2.00	1.74	1.57 to 1.93	< 0.001
2	637	421 (66.1%)	2.81	2.50 to 3.16	2.57	2.26 to 2.93	< 0.001
3+	616	505 (81.9%)	4.57	4.09 to 5.10	4.04	3.55 to 4.59	< 0.001
Exacerbations requiring A&E attendance or hospitalisation in baseline year							
0	4885	2302 (47.1%)	1	Ref			
1+	345	207 (60.0%)	1.49	1.29 to 1.71			
Antibiotics prescriptions in baseline year							
0	2659	895 (33.6%)	1	Ref			
1	1298	682 (52.5%)	1.81	1.64 to 2.00			
2	600	390 (65.0%)	2.6	2.31 to 2.93			
3+	673	542 (80.5%)	4.29	3.86 to 4.78			
OCS prescriptions in baseline year							
0	2108	769 (36.5%)	1	Ref			
1	992	454 (45.7%)	1.34	1.19 to 1.50	1.02	0.90 to 1.15	0.791
2	680	375 (55.1%)	1.75	1.55 to 1.98	1.14	1.00 to 1.30	0.053
3+	1450	911 (62.8%)	2.21	2.01 to 2.44	1.22	1.09 to 1.37	< 0.001
ICS medication possession ratio in baseline year							
≥ 70–< 80%	1559	728 (46.7%)	1	Ref			
≥ 80–< 90%	1049	491 (46.8%)	1	0.90 to 1.13	1	0.90 to 1.13	0.947
≥ 90–< 100%	1254	599 (47.7%)	1.05	0.94 to 1.17	0.99	0.88 to 1.10	0.804
≥100%	1368	691 (50.5%)	1.12	1.01 to 1.24	1.03	0.93 to 1.15	0.55
Time since first COPD diagnosis, year							
< 1	321	132 (41.1%)	1	Ref			
≥1–< 5	1360	607 (44.6%)	1.11	0.92 to 1.34			
≥ 5–< 10	1741	862 (49.5%)	1.29	1.07 to 1.55			
≥ 10–< 15	1017	502 (49.3%)	1.29	1.07 to 1.56			
≥ 15	639	330 (51.6%)	1.38	1.13 to 1.69			
Time on triple therapy, year							
< 1	896	386 (43.1%)	1	Ref			
≥1–< 3	1626	739 (45.4%)	1.1	0.97 to 1.24			
≥ 3–< 5	1234	596 (48.3%)	1.2	1.05 to 1.36			
≥ 5	1474	788 (53.4%)	1.38	1.22 to 1.55			

Only variables where univariate analysis gave a p-value < 0.05 are shown

### Secondary outcomes: exacerbations and pneumonia

In the outcome year 501/1046 (47.9%) of ICS cessation patients experienced at least one exacerbation, percentage that was almost identical to the 48.0% observed in control patients who remained on triple therapy (2008/4184). In both groups there were slightly more patients who experienced an exacerbation in the baseline year compared to the outcome year [542 (51.8%) in the ICS cessation group and 2233 (54.6%) in control patients].

The rate of exacerbations that were managed in primary care, resulting in A&E attendance or hospitalisation were slightly higher in the ICS cessation group when compared to the control group (Table 4). The univariate and multivariate comparisons are presented in Table 5. In multivariate analysis the number of episodes in the ICS cessation group was significantly higher [primary care incidence rate ratio (IRR) 1.33 (95% CI 1.10–1.60), p = 0.003; A&E attendance or hospitalisation IRR 1.72 (95% CI 1.03–2.86), p = 0.036, Table 5].

**Table 4:** Secondary outcomes in the ICS cessation and control groups in the 1-year outcome period

	Total No. 5230	Control No. 4184	ICS cessation No. 1046
Exacerbations			
Managed in primary care	0.86 (± 1.33)	0.86 (± 1.32)	0.90 (± 1.35)
Resulting in A&E attendance	0.09 (± 0.38)	0.09 (± 0.36)	0.11 (± 0.45)
Total exacerbations	0.96 (± 1.42)	0.94 (± 1.41)	1.01 (± 1.46)
Change in FEV_1_			
Baseline FEV1 (L)	1.39 (± 0.57)	1.36 (± 0.56)	1.49 (± 0.60)
Outcome FEV1 (L)	1.35 (± 0.58)	1.33 (± 0.58)	1.43 (± 0.60)
Annualised change in FEV1 (in mL)	− 25.1 (± 248.1)	− 18.8 (± 253.2)	− 48.8 (± 226.4)
Missing (n (%))	3777 (72.2%)	3035 (72.5%)	742 (70.9%)
CAT score in outcome year^a^			
< 10	183 (24.0%)	135 (23.9%)	48 (24.4%)
≥ 10	580 (76.0%)	431 (76.1%)	149 (75.6%)
Mean (SD)	16.6 (± 9.6)	16.8 (± 9.9)	16.2 (± 8.8)
Missing	4467 (85.4%)	3618 (86.5%)	849 (81.2%)
mMRC dyspnea score in outcome year^a^			
0–1	1345 (36.2%)	1072 (35.6%)	273 (38.7%)
2–4	2371 (63.8%)	1938 (64.4%)	433 (61.3%)
Mean (SD)	1.9 (± 1.0)	2.0 (± 1.0)	1.9 (± 1.0)
Missing	1514 (28.9%)	1174 (28.1%)	340 (32.5%)

^a^First score recorded following IPD is used. Values given are mean (SD)

**Table 5:** Univariate and multivariate analysis of the effects of ICS cessation compared to continuing triple therapy on secondary outcomes in the outcome year

	Univariate analysis			Multivariate analysis		
	IRR	95% CI	p-value	IRR	95% CI	p-value
Exacerbation rate						
Managed in primary care	1.05	0.95 to 1.17	0.330	1.33	1.10 to 1.60	0.003
Resulting in A&E attendance	1.24	0.95 to 1.62	0.109	1.72	1.03 to 2.86	0.036

	ß coef	95% CI	p-value	ß coef	95% CI	p-value
Change in FEV1	− 30.03	− 61.39 to 1.33	0.061	− 24.85	− 55.55 to 5.84	0.112

	OR	95% CI	p-value	OR	95% CI	p-value
CAT score						
≥ 10	0.97	0.67 to 1.43	0.884	1.02	0.60 to 1.76	0.936
mMRC dyspnea score						
≥ 2	0.88	0.74 to 1.04	0.129	0.77	0.54 to 1.10	0.161

**Fig. 3:**
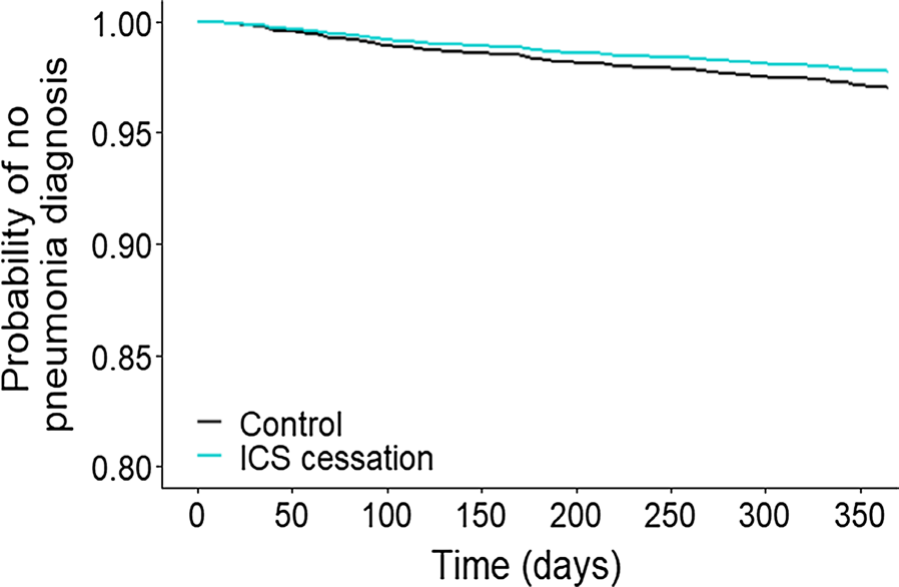
Plot of multivariate Cox proportional hazards model of time to first consultation for pneumonia in the 1-year outcome period. HR = 0.69 (95% CI 0.45 to 1.08; p = 0.108)

In the outcome year 24 (2.3%) ICS cessation patients had a consultation coded for pneumonia versus 52 (5%) in the baseline year. Among control patients 126 (3.0%) had a pneumonia in the outcome year compared to 101 (2.4%) in the baseline year. Having pneumonia in the baseline year increased the risk of having pneumonia in the outcome year; adjusting for this and age, the cessation of ICS was associated with a non-significantly reduced risk of having a consultation coded for pneumonia in the outcome year (HR 0.69 (95% CI 0.45–1.08), p < 0.108) (Fig. 3).

### Secondary outcomes: lung function and symptoms

In 304 (29.1%) ICS cessation patients and 1149 (27.5%) control patients who had FEV_1_ measurements recorded during both the baseline year and between 9 and 15 months post-IPD, the annualised change of FEV1 was not significantly different between groups (ICS cessation − 48.8 mL (SD: 226 mL) versus − 18.8 (SD: 253 mL) in control group (Table 4). Results of multivariate analysis adjusted for FEV_1_% predicted recorded at baseline and the total number of exacerbations in the outcome year showed non-significant beta − 24.85 (95% CI − 55.55 to 5.84), p = 0.112) (Table 5).

Using multivariate logistic regression analyses we investigated the odds of a CAT score ≥ 10 and the odds of a mMRC dyspnoea score ≥ 2 recorded at any point in the outcome year. Only 14.6% of patients had a CAT score recorded in the outcome year; of those, 75.6% of ICS cessation patients had a score ≥ 10 compared to 76.1% of control patients (Table 4). The odds of having a CAT score ≥ 10 in the 1-year outcome period were not significantly different in the ICS cessation group compared to the control group (adjusted odds ratio (OR) 1.02 (95% CI 0.60–1.76), p = 0.936, Tables 4 and 5). Similarly, 71.1% of patients had a mMRC score recorded, of those 61.3% of ICS cessation patients had a score ≥ 2 compared to 64.4% of control patients (adjusted OR 0.77 (95% CI 0.54–1.10), p = 0.161, Tables 4 and 5). The odds of either a CAT score ≥ 10 or mMRC score ≥ 2 was not significantly associated with the time from IPD to when the measurement was recorded or with the total number of exacerbations in the outcome year.

### Successful ICS withdrawal

**Fig. 4:**
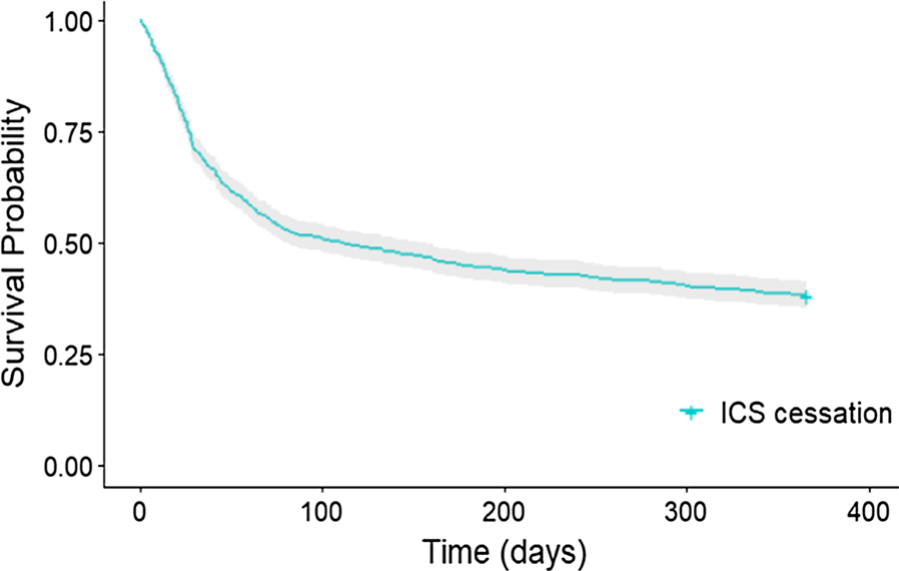
Time to reinitiation of ICS

In the outcome year 3516 (84%) control patients maintained a medication possession ratio ≥ 70% with a mean medication possession ratio of 103.9% (± 44.8), and 647 (61.9%) ICS cessation patients had reinitiated ICS. Among them, 60 (5.7%) reinitiated ICS within 7 days and 306 (29.3%) within 30 days (Fig. 4). Interestingly, only 139 cases (21.5%) where ICS was reinitiated had an exacerbation recorded prior to, or at the time of ICS reinitiation.

ICS cessation was considered successful if a patient did not have any exacerbations and did not reinitiate ICS treatment in the outcome year. 247 (23.6%) ICS cessation patients successfully stopped ICS, while the remaining 799 either restarted ICS or experienced at least one exacerbation in the outcome year. In a multivariate logistic regression model, the odds of successful ICS withdrawal were significantly reduced by having a blood eosinophil count ≥ 0.3 and by having more prescriptions of oral corticosteroids in the year prior to ICS cessation (Table 6).

**Table 6:** Determinants of successful ICS cessation

Variable	Total	Event		Univariate analysis				Multivariate analysis			
				HR	95% CI	p-value		HR	95% CI		p-value
Asthma diagnosis pre-baseline											
No	899	223 (24.8%)			Ref						
Yes	147	24 (16.3%)		0.59	0.37 to 0.94	0.026					
Blood eosinophil count											
< 0.1	99	27 (27.2%)			Ref						
≥ 0.1 to < 0.3	608	145 (23.8%)		0.84	0.52 to 1.35	0.462		0.76	0.47 to 1.27		0.282
≥ 0.3	182	31 (17.0%)		0.55	0.30 to 0.99	0.044		0.50	0.27 to 0.91		0.023
Exacerbations managed in primary care in baseline year											
0	535	171 (31.9%)			Ref						
1	258	55 (21.3%)		0.58	0.41 to 0.82	0.002					
2	119	12 (10.1%)		0.24	0.13 to 0.45	< 0.001					
3+	134	9 (6.7%)		0.15	0.08 to 0.31	< 0.001					
Antibiotic prescriptions in baseline year											
0	553	175 (31.6%)			Ref						
1	243	51 (20.9%)		0.57	0.40 to 0.82	0.002					
2	107	9 (8.4%)		0.20	0.10 to 0.40	< 0.001					
3+	143	12 (8.4%)		0.20	0.11 to 0.37	< 0.001					
OCS prescriptions in baseline year											
0	476	155 (32.5%)			Ref						
1	183	46 (25.1%)		0.70	0.47 to 1.02	0.064		0.70	0.45 to 1.05		0.090
2	116	13 (11.2%)		0.26	0.14 to 0.48	< 0.001		0.23	0.11 to 0.44		< 0.001
3+	271	33 (12.2%)		0.29	0.19 to 0.43	< 0.001		0.31	0.19 to 0.47		< 0.001

### Sensitivity analyses

In order to analyse the consistency of the findings, we performed some sensitivity analyses of the main outcome in different population of patients with COPD. The first analysis compared time to the first exacerbation in both groups of patients in those individuals who experienced 0 or 1 exacerbation the previous year and those with 2 or more. The second analysis evaluate the effect of ICS withdrawal independently in patients with either mild-moderate or severe-very severe COPD. The third analysis evaluated the risk in patients with a concomitant diagnosis of asthma. The fourth analysed the main outcome separately in patients with blood eosinophil counts below or above 300 cells/μL. The fifth analysed the risk of ICS withdrawal in patients with < 2 exacerbations and < 300 eosinophils/μL; and the final analysis investigated the risk of withdrawal excluding control patients with MPR < 70% and censoring ICS cessation patients who reinitiated ICS prior to their first exacerbation. All these analyses showed no significantly increased risk of exacerbation associated with ICS withdrawal (OR ranging from 0.954 to 1.08, p > 0.05 in all comparisons) (see Additional file 1^1^ Figures S2–S7).

## Discussion

The present study shows that patients with COPD followed in primary care in the UK did not have an increased risk of exacerbations after withdrawal of ICS as compared to those patients remaining on TT within 1-year of observation. The rates of exacerbations went down in the outcome year compared to the baseline year in both groups of patients; however, the adjusted incidence rate ratio was in favour of ICS continuation, in particular for the exacerbations resulting in secondary care. It is noteworthy that ICS cessation group had higher respiratory and pneumonia-coded consultations in primary care in the baseline year. The risk of reinitiating ICS or suffering an exacerbation in the withdrawal group was significantly increased in patients with blood eosinophil counts ≥ 300 cells/μL and in those having more prescriptions of oral corticosteroids in the year prior to ICS cessation. No significant differences in the changes in FEV1, CAT scores or mMRC dyspnoea score between patients who discontinued ICS and those who continued with TT were observed.

There is still a controversy about the rationale for use of ICS in COPD [16]. Current recommendations indicate that ICS should be given in combination with long-acting bronchodilators for patients with frequent or severe exacerbations and increased eosinophilic profile [1]. These patients represent only approximately one fourth to one third of the patients attending primary care, as shown in large studies that identified around 25% of patients with a frequent exacerbator phenotype [17, 18] and between 20 and 30% with a blood eosinophil count higher than 300 cells/mL [19]. However, several studies in different countries have shown an excessive use of ICS outside the current indications; a recent study in the UK showed that 13.7% of GOLD A and 26.2% of GOLD B received TT [7]; similarly, data from Switzerland observed a use of TT in 13.8% of GOLD A and 28.2% of GOLD B patients [6]. This excessive use of ICS in primary care may be explained, at least in part, by the difficulties in differentiating asthma from COPD or by the inadequate consideration of the history of exacerbations, the peripheral blood eosinophil counts or the risk of community-acquired pneumonia [20, 21].

Since the inadequate long-term use of ICS is associated with increased risk of side effects, it is important to identify patients in whom the risk/benefit ratio clearly supports the use of these drugs. Furthermore, in the case of patients inadequately treated with ICS, it is important to investigate the possible risk associated with the withdrawal of ICS.

Early trials investigating the risks associated with withdrawal of ICS provided conflicting results, basically due to small sample sizes, different inclusion criteria and, more importantly, due to insufficient or inadequate alternative treatments after ICS discontinuation [22]. The more recent randomised control trials (RCT) designed to investigate the risks associated with discontinuation of ICS compared patients on TT with those withdrawing ICS and continuing on dual bronchodilation as alternative. One trial included patients with severe airflow limitation (FEV1 < 50% predicted) and a history of at least one exacerbation during the year prior to enrolment [23]. ICS withdrawal did not lead to an increased number of COPD exacerbations compared to continued ICS users. ICS withdrawal led to a statistically significant decrease of lung function, which was not clinically relevant and a subsequent post-hoc analysis showed that the rate of decline of lung function was no different in patients who discontinued or in patients who continued ICS [24]. Another post-hoc analysis found that discontinuation of ICS was associated with an increased rate of moderate or severe exacerbations in the smaller subgroup of patients with eosinophil counts of ≥ 300 cells/μL or 4% or greater, whereas there was no difference in exacerbation rate in the remaining patients [25].

Another trial studied the effects of ICS abrupt withdrawal in COPD patients with an FEV1 50–80% predicted and a history of at least one exacerbation over the preceding year and who received TT for at least 6 months [26]. Similarly to the previous trial [24], no difference was observed in the rate of exacerbations after ICS withdrawal, and only a small reduction of FEV1 was observed in the ICS withdrawal group. Only in the 25% of patients with blood eosinophil count ≥ 300 cells/μL, the number of moderate and severe COPD exacerbations was significantly higher following ICS withdrawal [26].

These two large studies were the basis of the American Thoracic Society (ATS) guidelines, which indicates a conditional recommendation for ICS withdrawal for patients with COPD receiving TT if the patient has had no exacerbations in the past year [14]. Similarly, the European Respiratory Society (ERS) has issued a conditional recommendation for the withdrawal of ICS in patients with COPD without a history of frequent exacerbations and a strong recommendation not to withdraw ICS in patients who have a blood eosinophil count ≥ 300 eosinophils/μL, with or without a history of frequent exacerbations [15].

These guidelines are based on the results of RCTs and derived from well- characterized study populations, which may not always represent the general patient population [4]. Therefore, high quality observational studies including all types of patients attended in primary care are useful to complement the findings obtained in RCTs. In this context, our study has analysed data on 1046 patients who discontinued ICS, matched 1:4 for time on TT with 4184 controls who continued on TT over a period of 1 year of observation. As in the majority of studies in primary care, more than three quarters (76.1%) of patients were infrequent exacerbators, and the results showed that patients who had withdrawn ICS did not have an increased risk of exacerbation, without any differences in changes in FEV1, CAT scores and mMRC degree of dyspnoea.

Our results are concordant with those obtained in other previous observational studies. Rossi et al. [27] investigated the results of withdrawal of ICS in real life in COPD patients with FEV1 > 50% and less than two exacerbations per year. Their results showed no differences in lung function, symptoms and exacerbations between patients who withdrew or continued ICS within a 6-month observation period. In an observational study conducted in primary and secondary care in Germany [28], patients who discontinued ICS at study entry did not have a different risk of exacerbations over a 2-year observation period. Using data from the Clinical Practice Research Datalink (CPRD) in the UK, Oshagbemi et al. [29] did not observe any increase in risk of moderate or severe exacerbations or mortality in a population of + 40,000 COPD patients in primary care irrespective of the blood eosinophil counts. Finally, in a retrospective study on the effect of withdrawal of ICS after hospitalisation for COPD in Japan, Jo et al. [30] observed a reduced incidence of re-hospitalisation for COPD exacerbations or death in patients who withdraw ICS.

Although ICS withdrawal was not associated with increased risk of exacerbation for the whole group, the rate of exacerbations was slightly higher in the withdrawal group; these findings suggest that there might be a small subgroup of patients who are susceptible to discontinuation and had repeated episodes during the outcome year. Our definition of successful discontinuation of ICS included those patients who did not experience any exacerbation and did not reinitiate ICS during the outcome year. It is possible that not all reinitiations of ICS were due to clinical deterioration. In fact, only 21.5% cases of reinitiation had an exacerbation recorded prior to, or at the time of ICS reinitiation (see Figs. 2 and 4). Furthermore, having an exacerbation after ICS withdrawal may not always mean an unsuccessful discontinuation, because the patient may have also had one or more exacerbations the previous year while on ICS. In any case, with this conservative definition, the risk of unsuccessful ICS withdrawal was significantly increased only in patients with blood eosinophil counts ≥ 300 cells/μL and in those having more prescriptions of oral corticosteroids in the year prior to ICS cessation. These results support the ATS and particularly the ERS guidelines that recommend discontinuation of ICS in patients without a history of frequent exacerbations and blood eosinophils < 300 cells/μL [14, 15].

Despite the published studies and recommendations, ICS withdrawal is very infrequent in real life in primary care [31, 32]. In our study, only 2% of patients on TT discontinued ICS during 1 year, which is similar to the 2% to 3.5% rates of discontinuation of ICS observed between 2014 and 2018 in another large primary care study in the UK [33]. However, other studies have observed higher rates, such as the 15% observed in a population-based study on + 34,000 patients on TT in primary care in Spain [34] or the 16% in Korea [35].

A reduction in the risk of pneumonia has been demonstrated after discontinuation of ICS in large population-based studies, and this risk drops especially during the first 3 months after discontinuation [36]. We have also observed a reduced risk of pneumonia in patients who withdraw ICS, although the reduction was not statistically significant probably due to the low number of events registered.

Our study has some limitations, inherent to the observational design: Firstly, although patients who discontinued ICS were milder in terms of airflow obstruction, they had more frequent exacerbations the baseline year compared to those who continued ICS, and could, therefore be more prone to suffer exacerbations in the outcome year, biasing the results against the safety of withdrawal. Secondly, although the analysis controlled for confounders, the observational design is not completely free from bias. Thirdly, our observations on the evolution of CAT scores, FEV1 and mMRC were based on the small subgroup of patients with at least two measurements; however, the results of all these variables were consistent with each other.

In summary, discontinuation of ICS from TT is still very infrequent in primary care. We have not observed an increased risk of exacerbations after discontinuation of ICS in our cohort of mainly infrequent exacerbators; However, there was an increased risk of unsuccessful ICS discontinuation in patients with frequent exacerbations and high blood eosinophil levels, which resulted in an increased rate of exacerbation for the overall withdrawal group. Our results support the recommendations that withdrawal of ICS should be considered in patients with COPD without a history of frequent exacerbations and low blood eosinophil counts.

## Conclusions

In this primary care population of patients with COPD, composed mostly of infrequent exacerbators, discontinuation of ICS from TT was not associated with an increased risk of exacerbation; however, according with current guidelines, the subgroup of patients with more frequent courses of oral corticosteroids in the past year and high blood eosinophil counts should not be withdrawn from ICS.

## Supplementary Information

**Additional file 1: Figure S1**. Kaplan–Meier plot of time to leaving the database. **Figure S2.** Time to first exacerbation in those with 0 or 1 exacerbation and 2+ exacerbations during the baseline year. **Figure S3.** Time to first exacerbation in those with mild or moderate COPD and those with severe or very severe COPD, based on GOLD categories. **Figure S4.** Time to first exacerbation in those with a diagnosis of asthma prior to the baseline year and to those who have never received an asthma diagnosis. **Figure S5. **Time to first exacerbation in those with a baseline blood eosinophil level < 0.3 and those a level ≥ 0.3. **Figure S6.** Time to first exacerbation in those with 0–1 baseline exacerbation AND a baseline blood eosinophil level < 0.3. **Figure S7.** A) Excluding control patients with MPR < 70% and censoring ICS cessation patients who reinitiated ICS prior to their first exacerbation. **Table S1.** Time on triple therapy prior to IPD after matching.

## Data Availability

The datasets generated and/or analysed during the current study are not publicly available.

## Abbreviations

ADEPT: Anonymised Data Ethics & Protocol Transparency
A&E: Accident and emergency
ATS: American Thoracic Society
BMI: Body mass index
CAT: COPD Assessment Test
CI: Confidence interval
CPRD: Clinical Practice Research Datalink
COPD: Chronic obstructive pulmonary disease
EUPAS: European Network of Centres for Pharmacoepidemiology and Pharmacovigilance
ERS: European Respiratory Society
FEV_1_: Forced expiratory volume in 1 s
FVC: Forced vital capacity
GOLD: Global strategy for Obstructive Lung Disease
GP: General practice
HR: Hazard ratio
ICS: Inhaled corticosteroid
IPD: Index prescription date
IRR: Incidence rate ratio
LABA: Long-acting beta-2 agonist
LAMA: Long-acting antimuscarinic agent
mMRC: Modified Medical Research Council dispnea scale
MPR: Medication possession ratio
OPCRD: Optimum Patient Care Research Database
OR: Odds ratio
REG: Respiratory Effectiveness Group
RCT: Randomised clinical trial
TT: Triple therapy
UK: United Kingdom
