# Supplementary material for: Withdrawal of inhaled corticosteroids versus continuation of triple therapy in patients with COPD in real life: observational comparative effectiveness study
Source: Respir Res. 2021 Jan 21;22:25. doi: 10.1186/s12931-021-01615-0 (PMC7818945; doi:10.1186/s12931-021-01615-0)
Supplement: Supplementary file 1 — Additional file 1: Figure S1. Kaplan–Meier plot of time to leaving the database. Figure S2. Time to first exacerbation in those with 0 or 1 exacerbation and 2+ exacerbations during the baseline year. Figure S3. Time to first exacerbation in those with mild or moderate COPD and those with severe or very severe COPD, based on GOLD categories. Figure S4. Time to first exacerbation in those with a diagnosis of asthma prior to the baseline year and to those who have never received an asthma diagnosis. Figure S5. Time to first exacerbation in those with a baseline blood eosinophil level < 0.3 and those a level ≥ 0.3. Figure S6. Time to first exacerbation in those with 0–1 baseline exacerbation AND a baseline blood eosinophil level < 0.3. Figure S7. A) Excluding control patients with MPR < 70% and censoring ICS cessation patients who reinitiated ICS prior to their first exacerbation. Table S1. Time on triple therapy prior to IPD after matching. [file 12931_2021_1615_MOESM1_ESM.docx]

**Supplementary material**

| \| **Supplementary table 1.** Time on triple therapy prior to IPD after matching \| \| --- \| \| \|  \| \| \| \| \| \| --- \| --- \| --- \| --- \| --- \| \|  \|  \| \| \| \| \| **Years** \| **Total No. 5,230** \| **Control No. 4,184** \| **ICS cessation No. 1,046** \| **p-value** \| \| \| < 1 \| 896 (17.1%) \| 728 (17.4%) \| 168 (16.1%) \| < 0.001 \| \| \| ≥1 <4 \| 2,245 (42.9%) \| 1,785 (42.7%) \| 460 (44.0%) \|  \| \| \| ≥4 <7 \| 1,481 (28.3%) \| 1,225 (29.3%) \| 256 (24.5%) \|  \| \| \| ≥7 <10 \| 490 (9.4%) \| 363 (8.7%) \| 127 (12.1%) \|  \| \| \| ≥10 \| 118 (2.3%) \| 83 (2.0%) \| 35 (3.3%) \|  \| \| \| \|  \|  \| **Supplementary figure 1**. Kaplan-Meier plot of time to leaving the database \| \| --- \| \| 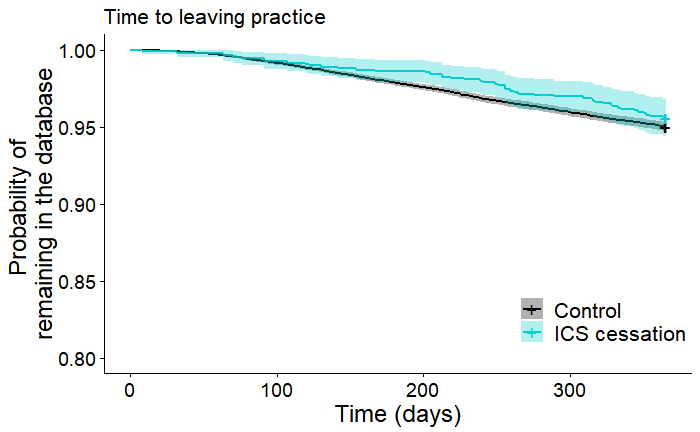 \|   **Footnote:** After a year 4.4 % (48/1094) of ICS cessation patients had left the practice compared to 5.0 % (1127/22704) of control patients, which was not significantly different (p= 0.38)  **Supplementary figure 2a**   \| Time to first exacerbation in those with 0 or 1 exacerbation and 2+ exacerbations during  the baseline year. \| \| \| --- \| --- \| \| 0 or 1 baseline exacerbation \|  \| \| 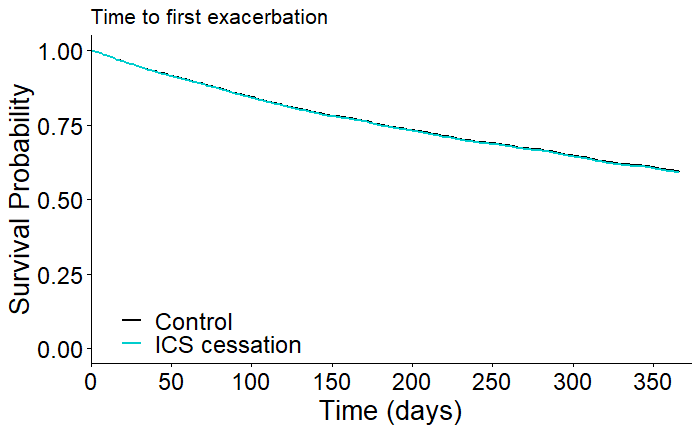 \|  \| \| After 1 year an exacerbation was experienced by: 38.4% (295/769) of ICS cessation patients and 39.7% (1228/3090) of control patients  HR 1.01 (95% CI 0.88-1.16), p=0.901  **Supplementary figure 2b**  2+ baseline exacerbations \|  \| \| 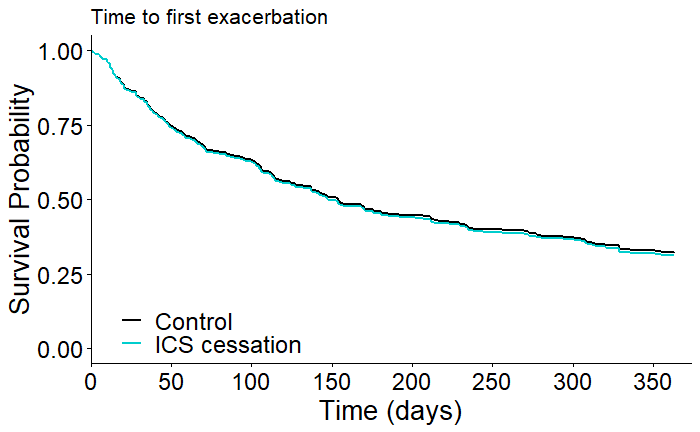 \|  \| \| After 1 year an exacerbation was experienced by: 74.4% (206/277) of ICS cessation patients and 71.3% (780/1094) of control patients  HR 1.02 (95% CI 0.75-1.39), p= 0.878 \|  \| \|  \|  \|  \| **Supplementary figure 3a**  Time to first exacerbation in those with mild or moderate COPD and those with severe or very severe COPD, based on GOLD categories. \| \| \| --- \| --- \| \| Mild and moderate patients \|  \| \| 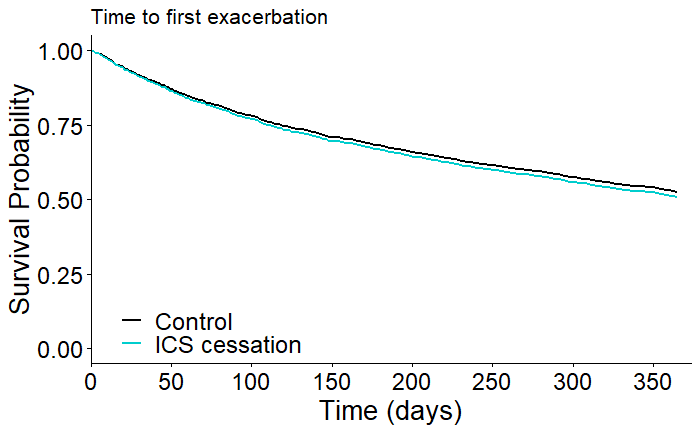 \| After 1 year an exacerbation was experienced by:  46.7% (300/643) of ICS cessation patients  46.5% (955/2055) of control patients  HR 1.06 (95% CI 0.92-1.22), p=0.432 \| \| **Supplementary figure 3b**  Severe and very severe patients \|  \| \| 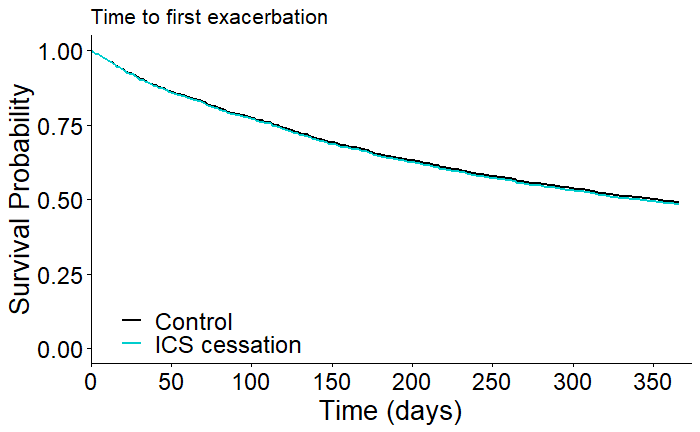 \| After 1 year an exacerbation was experienced by:  50.4% (176/349) of ICS cessation patients  50.9% (872/1712) of control patients  HR 1.02 (95% CI 0.87-1.21), p=0.783 \|  \| **Supplementary figure 4a**  Time to first exacerbation in those with a diagnosis of asthma prior to the baseline year and to those who have never received an asthma diagnosis. \| \| \| --- \| --- \| \| History of asthma \|  \| \| 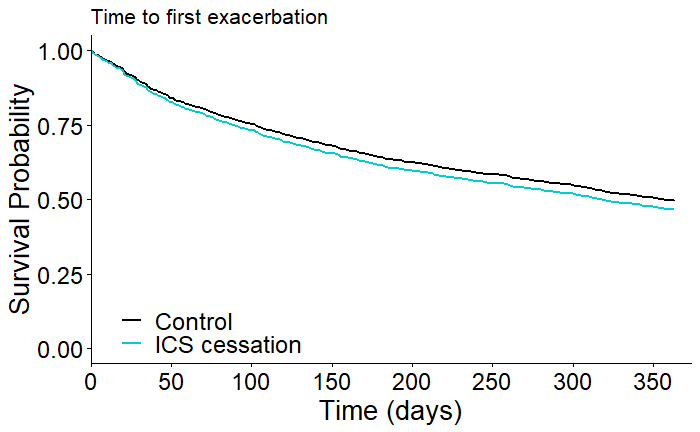 \| After 1 year an exacerbation was experienced by:  54.4% (80/147) of ICS cessation patients  50.2% (565/1126) of control patients  HR 1.06 (95% CI 0.84-1.34), p=0.613 \| \| **Supplementary figure 4b**  No history of asthma \|  \| \| 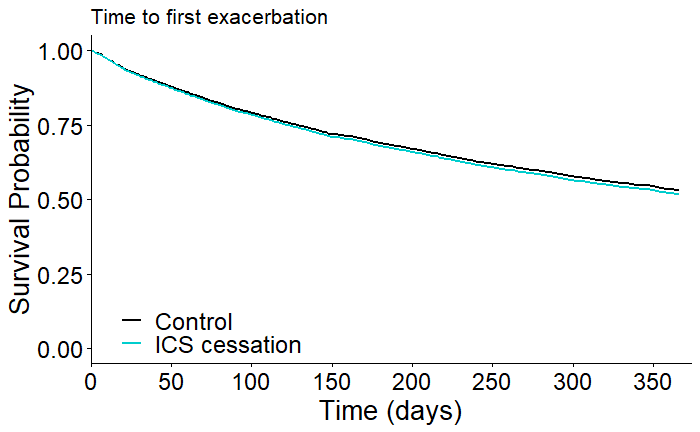 \| After 1 year an exacerbation was experienced by:  46.7% (421/899) of ICS cessation patients  47.2% (1443/3058) of control patients  HR 1.04 (95% CI 0.93-1.17), p=0.462 \|   **Supplementary figure 5a**   \| Time to first exacerbation in those with a baseline blood eosinophil level <0.3 and those a level ≥ 0.3. \| \| \| --- \| --- \| \| Blood eosinophils <0.3 \|  \| \| 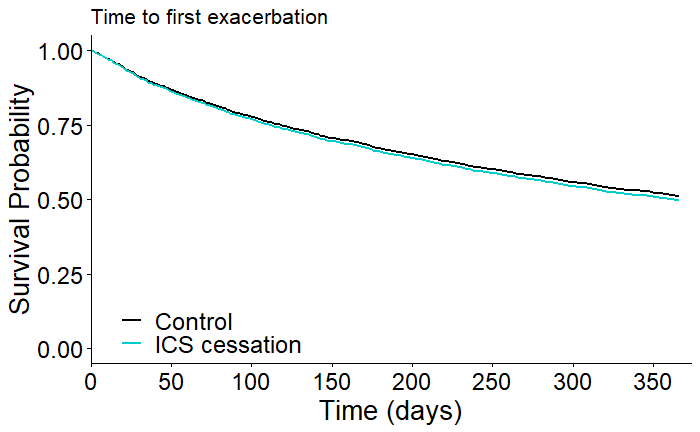 \| After 1 year an exacerbation was experienced by:  49.1% (347/707) of ICS cessation patients  49.0% (1278/2610) of control patients  HR 1.05 (95% CI 0.93-1.18), p=0.453 \| \| **Supplementary figure 5b**  Blood eosinophils ≥0.3 \|  \| \| 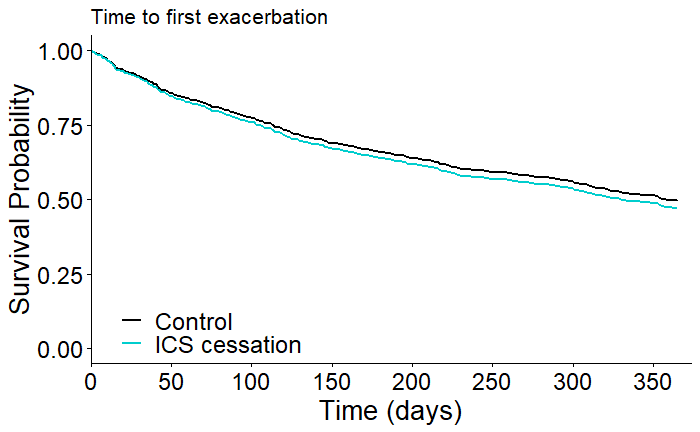 \| After 1 year an exacerbation was experienced by:  51.1% (93/182) of ICS cessation patients  50.4% (397/788) of control patients  HR 1.08 (95% CI 0.86-1.37), p=0.503 \|   **Supplementary figure 6**   \| Time to first exacerbation in those with 0-1 baseline exacerbation AND a baseline blood eosinophil level <0.3. \| \| \| --- \| --- \| \| 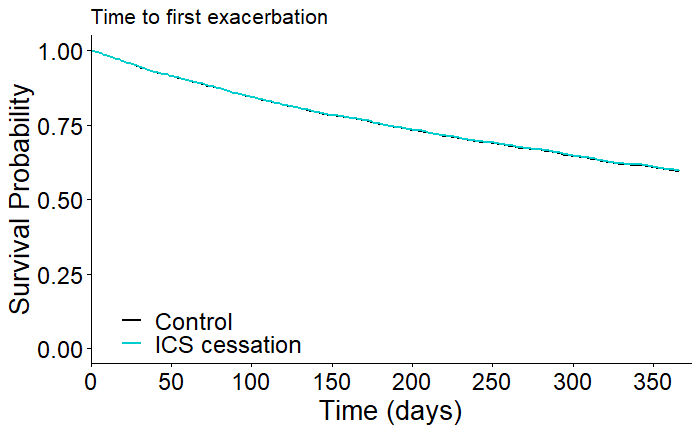 \| After 1 year an exacerbation was experienced by:  39.6% (205/518) of ICS cessation patients  40.8% (781/1916) of control patients  HR 1.00 (95% CI 0.85-1.16), p=0.954 \| \|  \|  \|   **Supplementary figure 7**   \| A) Excluding control patients with MPR<70% and censoring ICS cessation patients who reinitiated ICS prior to their first exacerbation \| \| \| \| \| \| \| \| \| \| \| --- \| --- \| --- \| --- \| --- \| --- \| --- \| --- \| --- \| --- \| \| 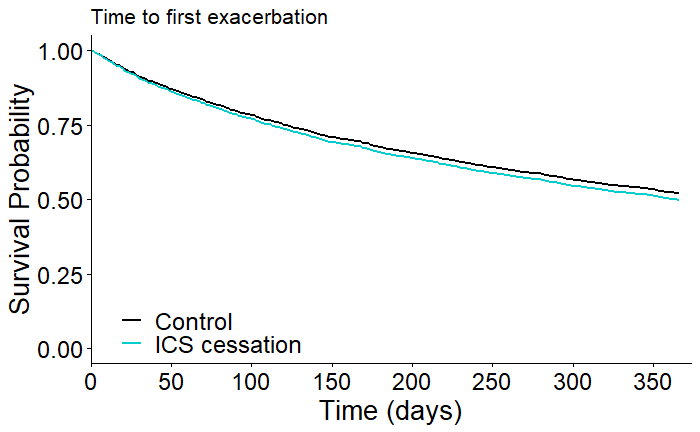 \| \| \| \| \| \| \| \| \| \| \| ICS cess. \| 1046 \|  \| 418 \|  \| 319 \|  \| 272 \|  \| 247 \| \| Control \| 3516 \|  \| 2783 \|  \| 2322 \|  \| 1990 \|  \| 1813 \| \|  \| 0 \|  \| 100 \|  \| 200 \|  \| 300 \|  \| 365 \| \|  \| Time (days)  HR 1.07 (95% CI 0.94-1.22), p=0.281 \| \| \| \| \| \| \| \| \| |
| --- | --- | --- | --- | --- | --- | --- | --- | --- | --- | --- | --- | --- | --- | --- | --- | --- | --- | --- | --- | --- | --- | --- | --- | --- | --- | --- | --- | --- | --- | --- | --- | --- | --- | --- | --- | --- | --- | --- | --- | --- | --- | --- | --- | --- | --- | --- | --- | --- | --- | --- | --- | --- | --- | --- | --- | --- | --- | --- | --- | --- | --- | --- | --- | --- | --- | --- | --- | --- | --- | --- | --- | --- | --- | --- | --- | --- | --- | --- | --- | --- | --- | --- | --- | --- | --- | --- | --- | --- | --- | --- | --- | --- | --- | --- | --- | --- | --- | --- | --- | --- | --- | --- | --- | --- | --- | --- | --- | --- | --- | --- | --- | --- | --- | --- | --- | --- | --- | --- | --- | --- | --- | --- | --- | --- | --- | --- | --- | --- | --- | --- | --- | --- | --- | --- | --- | --- | --- | --- | --- | --- | --- | --- | --- | --- | --- | --- | --- | --- | --- | --- | --- | --- | --- | --- | --- | --- | --- | --- | --- | --- | --- |
